# Changes in microbial community structure of bio‐fouled polyolefins over a year‐long seawater incubation in Hawai'i

**DOI:** 10.1111/1758-2229.13283

**Published:** 2024-07-29

**Authors:** Elizabeth Connors, Laurent Lebreton, Jeff S. Bowman, Sarah‐Jeanne Royer

**Affiliations:** ^1^ Scripps Institution of Oceanography La Jolla California USA; ^2^ Center for Marine Debris Research Hawaii Pacific University Waimānalo Hawaii USA; ^3^ The Ocean Cleanup Foundation Rotterdam The Netherlands

## Abstract

Plastic waste, especially positively buoyant polymers known as polyolefins, are a major component of floating debris in the marine environment. While plastic colonisation by marine microbes is well documented from environmental samples, the succession of marine microbial community structure over longer time scales (> > 1 month) and across different types and shapes of plastic debris is less certain. We analysed 16S rRNA and 18S rRNA amplicon gene sequences from biofilms on polyolefin debris floating in a flow‐through seawater tank in Hawai'i to assess differences in microbial succession across the plastic types of polypropylene (PP) and both high‐density polyethylene (HDPE) and low‐density polyethylene (LDPE) made of different plastic shapes (rod, film and cube) under the same environmental conditions for 1 year. Regardless of type or shape, all plastic debris were dominated by the eukaryotic diatom *Nitzschia*, and only plastic type was significantly important for bacterial community structure over time (*p* = 0.005). PE plastics had higher differential abundance when compared to PP for 20 bacterial and eight eukaryotic taxa, including the known plastic degrading bacterial taxon *Hyphomonas* (*p* = 0.01). Results from our study provide empirical evidence that plastic type may be more important for bacterial than eukaryotic microbial community succession on polyolefin pollution under similar conditions.

## Graphical abstract

While the presence of marine microbial communities on plastic debris is well documented, the succession of communities over long‐time scales (>1 month) is less certain. We incubated three shapes and types of plastic (LDPE, HDPE and PP) in a flow through seawater incubation tank for one year to assess differences in community structure. Diatoms dominated all types of plastic debris and bacterial communities differed across plastic types over the experiment.

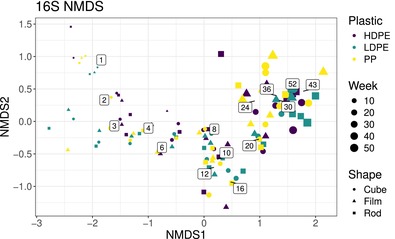

## INTRODUCTION

The most dominant commercial polyolefins—positively buoyant plastic polymers—are low‐density polyethylene (LDPE), high density polyethylene (HDPE) and polypropylene (PP) with a combined global production of over 150 million tons per year (Yuan et al., 2022). Up to 5% of plastic polymers produced per year are not recycled and is input inadvertently into the ocean where it forms a major fraction of marine debris (Jambeck et al., 2015). Mechanical breakdown and chemical alteration of plastic debris in the marine environment facilitates the colonisation of plastic debris by microorganisms—including bacteria, fungi and single‐celled eukaryotes—resulting in a biofilm known as the ‘plastisphere’ (Zettler et al., 2013; Zhai et al., 2023). While on larger debris these biofilms can include macro‐organisms like barnacles and hydroids, microbial colonisation occurs on all plastic debris including the smaller and more prolific microplastics (Wright et al., 2020).

Species composition of the ‘plastisphere’ is primarily dependent on time spent in the photic marine environment. Seawater incubation experiments have indicated ‘early stage’ colonisation is marked by stochastic colonisation and then rapid proliferation of biodegrading microbes. A prolific early bacterial coloniser of plastic debris is *Hyphomonas*, which can degrade polyethylene terephthalate and is typically found in hydrocarbon‐enriched soils (Denaro et al., 2020; Yakimov et al., 2005). Within a few weeks in the marine environment, the plastisphere is then dominated by a non‐biodegrading eukaryotic community (typically diatoms) and their associated bacteria (Datta et al., 2016; Wright et al., 2020). Known bacterial diatom‐associates in the plastisphere include *Rhodobacterales* and *Roseobacter*, which are important in biofilm formation (Amaral‐Zettler et al., 2020; Zhai et al., 2023).

It is clear yet if this pattern of first ‘early stage’ biodegrading bacterial succession and then ‘late‐stage’ eukaryotic stability is consistent across all shapes and types of polyolefins under similar conditions (Erni‐Cassola et al., 2020; Wallbank et al., 2022). However, as most incubation studies are shorter than 1 month, it is not clear if ‘late‐stage’ eukaryotic stability persists over months of seasonal change or across years spent on plastic debris in the marine environment. There is evidence that seasonal change alters marine plastisphere community structure in the North Sea, especially for bacteria (Oberbeckmann et al., 2014), but it is unclear if this pattern exists in more temperate climates such as coastal Hawai'i.

While time spent in the marine environment is the dominant driver of biofilm communities, plastic type is also important for shaping community structure across environmental gradients (Caruso, 2020; Oberbeckmann et al., 2017). Plastic type and shape greatly impact the rate of physical and chemical degradation of marine debris. For instance, in a marine setting, LDPE film degraded more quickly than other shapes and types of polyolefin (Chamas et al., 2020), and wave action and UV light caused cracking and embrittlement more quickly for thin shapes of plastic debris (Ivar do Sul & Costa, 2014). Plastic type determines how quickly chemical bonds change from simple carbon chains into those with carbonyl groups during degradation (Fotopoulou & Karapanagioti, 2017). An increase in carbonyl bonds makes plastic debris a more attractive substrate for microbial colonisation (Bank, 2022). It is presently unclear if the variable rate of degradation across plastic shapes and types is what drives the differences found in microbial community structure data, as few studies exist comparing plastic shape/type degradation and microbial colonisation under the same conditions.

Here, we use both bacterial (16S rRNA sequencing) and eukaryotic (18S rRNA sequencing) amplicon sequences to monitor community succession on polyolefins floating in a seawater flow‐through tank in Hawai'i. We first compared changes in community structure to environmental conditions, including seawater temperature, PAR, and oxygen over the year‐long experiment. We then divide plastics into two different types (PE and PP plastics) and compared the differential relative abundance of microbes across these two plastic types. We designed our study to improve our understanding of patterns in microbial succession which allowed us to examine the potential for biological degradation across different shapes and types of polyolefins over the course of a year.

## MATERIALS AND METHODS

### Plastic types and shape dimensions

Consumer grade HDPE (#2, TIPELIN BB620‐17), LDPE (#4, BRALEN RB 03–23) and PP (#5, TIPLEN H681 F) were obtained from MOL Petrochemicals Plc. (Budapest, Hungary) for the study. The LDPE contained a known antioxidant package of Irganox B215FF blended in at 0.15 wt% by IMSYS Engineering Service Ltd. (Budapest, Hungary) and remaining resins contained a proprietary antioxidant package. The three shapes selected were based on common shapes found in the environment and easily possible to make with a high degree of replicability. The shapes were the ones of rod (cylindrical shape), cube and film and were created with a LT 26–44 twin screw extruder (Labtech Engineering Company Ltd., Thailand) with filament‐die and either cut to shape with a granulating machine (rod and cube) or a paper cutting machine (film). Dimensions were chosen to maintain a consistent surface area across shapes, with the average dimensions of 3.45 × 3.08 × 3.20 mm for cube, 7.06 × 6.47 × 0.112 mm for film and 15.12 mm length with a 2.13 mm diameter for rod.

### Experimental design

Replicates of each plastic type and shape such as LDPE rod, LDPE cube, LDPE film with nine total sample types were incubated within nine separate flow‐through containers in a 6000 L outdoor flow‐through seawater tank at Hawai'i Pacific University (Oceanic Institute, Waimānalo, HI) for 1 year from April 15, 2021, to April 14, 2022 (see Royer et al., in prep). The tank was filled to a depth of 1.02 m with circulated seawater from a nearshore intake at Makapu'u Beach (15 m depth, coordinates 21.316376 N−157.663950 W) with a flow rate of 4.91 ± 3.66 L min^−1^. Intake water was filtered with a combination of filters, including a ~1 mm pore size medium density Matala filter to remove solid waste (Pentair), activated carbon media pads to remove any potential organic pollutants that would confound the results of the study (Fluval) and a biofilter (Sweetwater SWX Bio‐Media) to oxygenate seawater to saturation. Samples were taken for DNA analysis 15 times throughout the year‐long incubation: weekly for the first 4 weeks, every other week until week 12, every 4 weeks until week 24, and finally at weeks 30, 36, 43 and 52. Samples of each polymer type and shape for the medium size particle were chosen randomly from those floating on the surface only of the seawater tank. Given that the particles were small enough the whole particles were used in DNA extraction and added directly to lysis buffer and frozen at −80°C until DNA extraction and sequencing. Although most of the plastics in our experiment remained floating, a minority of the plastics sank to the bottom of our flow through experimental tank during incubation. At week 16 and week 24, opportunistic samples were taken from the bottom of the seawater tank (1.02 m depth), a majority of which were film (66%) in order to compare floating plastic and sunken plastic communities.

### Environmental data

Environmental data were measured for the duration of the experiment in high resolution with a suite of sensors mounted on the side of the seawater tank at the water surface. Dissolved oxygen (CS511 sensor, Campbell Scientific; Logan, UT), water temperature (ST‐100 sensor, Apogee Instruments; Logan, UT), air temperature (ST‐110 sensor with TS‐100 fan‐aspirated radiation shield, Apogee Instruments), and photosynthetically active radiation (PAR, SQ‐500 sensor with spectral range 412–682 (±5) nm, Apogee Instruments) measurements were recorded continuously at 1 s intervals using a CR6 datalogger equipped with Wi‐Fi transmittance (Campbell Scientific) and Loggernet software version 4.2.6 (Campbell Scientific). This high‐resolution data was limited to daytime measurements and averaged leading up to each of the 15 sampling days for DNA to demonstrate the cumulative environmental change leading up to each of the 14 DNA sampling days after time 0.

### DNA collection, extraction and amplicon sequencing

For the DNA samples, a plastic sample was randomly selected from each plastic type and shape and stored at −80°C until extraction. Microbial DNA was extracted from each plastic using the KingFisher™ Flex Purification System and MagMax Microbiome Ultra Nucleic Acid Extraction Kit (ThermoFisher Scientific, Waltham, Massachusetts, USA). Extracted DNA was sent to Argonne National Laboratory for amplicon library preparation and sequencing using the Illumina MiSeq platform with the primers 515F and 806R for 16S rRNA gene sequencing (Walters et al., 2016), 1380F and 1505R for 18S rRNA gene sequencing (Amaral‐Zettler et al., 2009), and a 2 × 151 bp library architecture.

### Processing microbial community structure data with paprica 0.7.1

Illumina reads were filtered, denoised and merged with DADA2 (Callahan et al., 2016) and then analysed with paprica v0.7.1 (Bowman & Ducklow, 2015). Paprica utilises phylogenetic placement with Gappa (Czech et al., 2020) EPA‐ng (Barbera et al., 2019) and Infernal (Nawrocki & Eddy, 2013), and RefSeq to place query reads on a reference tree constructed from the full‐length 16S rRNA genes from all completed genomes in GenBank (Haft et al., 2018) or PR2 4.13.0 for 18S rRNA genes (Guillou et al., 2013). All unique reads were assigned to internal branches or terminal branches on the reference tree. Once assigned, unique reads that were assigned as metazoan mitochondria or chloroplasts were omitted, as well as any reads that only appeared once.

### Statistical analyses

All analyses were performed using R Statistical Software (R, 2021). The output table of unique reads from paprica was used to analyse changes in community structure over time, plastic shape, and plastic type via alpha and beta diversity analysis. For our alpha diversity calculation, Shannon diversity of absolute abundances for a subset (500 ASVs) of each sample were calculated (via the diversity() function) with the vegan package (Oksanen et al., 2022). The analysis of covariance (ANCOVA) in Shannon diversity over time, plastic shape and plastic type was run with the software package cars (Fox & Weisberg, 2019), and post hoc e‐means with the package rstatix (Kassambara, 2023).

For our beta diversity analysis, unique reads were first cumulative sum scaled to normalise for sampling depth across samples with the R package metagenomeSeq (Paulson et al., 2013). Then, non‐metric multidimensional scaling (NMDS) of Bray–Curtis distance and data dispersion (via the betadisper() function) of the relative abundance table were conducted with the vegan package (Oksanen et al., 2022). Post‐hoc analysis of variance in Bray–Curtis distances across time, plastic shape, plastic type and environmental variables were conducted with pairwiseAdonis (Martinez Arbizu, 2020). Heatmaps were created with the package pheatmap (Kolde, 2019). The package deseq2 (Love et al., 2014) was used to determine differential abundance of unique reads at each time point. As deseq2 analysis requires binary sample categorisation (i.e., x vs. y), we compared PP samples to PE samples (LDPE and HDPE).

## RESULTS AND DISCUSSION

In our flow‐through seawater tank experiment, we saw significant differences in bacterial and eukaryotic community structure over a year of observations. The differences in bacterial community structure were more pronounced than eukaryotic community structure overall, with notable changes to the community over time, and more significantly different bacterial taxa across plastic type, especially in the PE versus PP differential abundance analysis.

### Environmental results

Previous works have shown that microbial diversity on plastic debris is highly dependent on seasonal environmental conditions in other ocean regions (Oberbeckmann et al., 2014; Zhai et al., 2023), as it was in our study. The daylight averages of PAR had a total range of 600–1000 μmol m^−2^ s^−1^, water temperature a range of 26°C–30°C and air temperature a range of 25°C–29°C over the year‐long experiment. The averages of all three measurements were elevated from June to September 2021 (week 8 through week 24, Figure S1A,B). Average dissolved oxygen had a range of 8–10 mg L^−1^ during the experiment. Oxygen remained near 9 mg L^−1^ until September (week 24), was highest in November (week 30, increasing to 13.5 mg L^−1^) and remained below 10 mg L^−1^ after January 2022 (week 40, Figure S1B). Our measurements of PAR, air temperature and water temperature followed expected seasonal trends for coastal Hawai'i, where temperature and PAR are highest from May to October and water temperature is highest in September (NOAA, 2023). These seasonal differences corresponded to changes in the microbial communities, especially in bacterial community structure over time.

### Bacterial community structure

**FIGURE 1:**
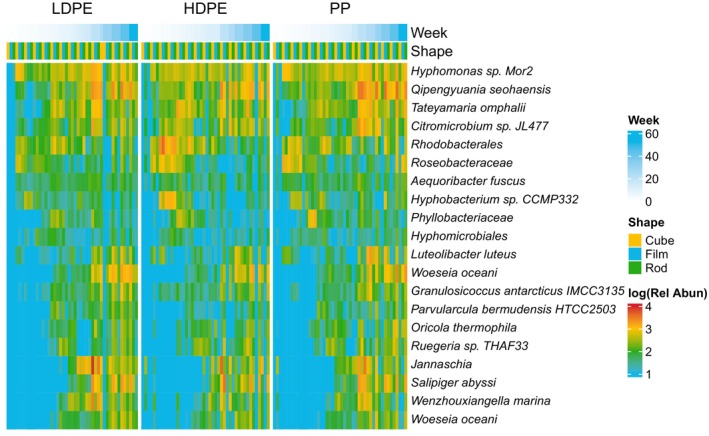
Heatmap of relative abundance (Rel Abun) of top 20 16S (bacteria and archaea), rRNA ASVs divided by polymer type (low‐density polyethylene (LDPE), high‐density polyethylene (HDPE) and polypropylene (PP)), shape (cube, film and rod) and ordered by week during the experiment from April 2021 to April 2022 in Hawai'i, USA.

**FIGURE 2:**
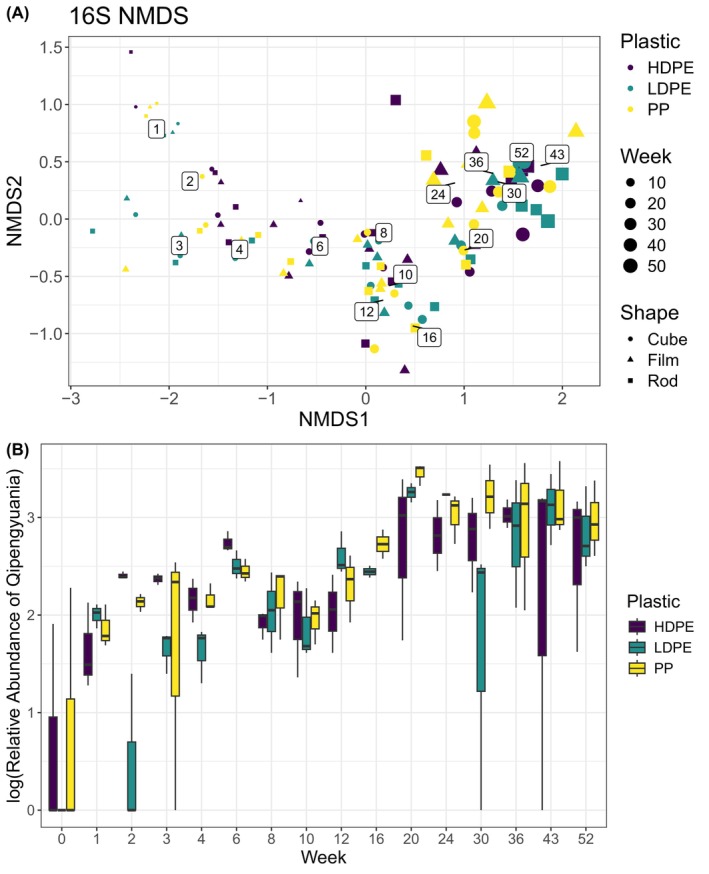
(A) Non‐metric multidimensional scaling (NMDS) of Bray–Curtis distance and (B) log(relative abundance) of the second most dominant taxa *Qipengyuania* over the year, which increased over the study period. NMDS stress was 0.10, and labelled numbers are average values for week sampled. Community structure was not significantly different over time or shape but was for plastic type (ADONIS *p* for time = 0, shape = 0.95 and plastic type = 0.05) week during the experiment from April 2021 to April 2022 in Hawai'i, USA.

Bacterial community structures (16S rRNA sequences) were dominated by the genera *Hyphomonas* and *Qipengyuania* across all plastic shapes and types (Figure 1). Shannon diversity index for bacterial community structure had a mean of 3.2 and was not significantly different across plastic type or shape (SD = 0.62, ANCOVA *p* type = 0.81 shape = 0.61). The second most dominant taxa *Qipengyuania* increased threefold in relative terms over the study period for all plastic types and shapes (Figure 2B). From April to June 2021 (week 0–8), there was an increase in the copiotrophic taxa *Rosebacter* and *Rhodobacterales*. Later in the experiment, 16S reads were dominated by other chemotrophic taxa, including *Tateyamaria* and *Woeseia* (Figure 2A,B).

In our NMDS analysis, Bray–Curtis distances of relative abundance did have significantly different compositions across time and polymer type (ADONIS *p* for time = 0.05, shape = 0.15 and polymer type = 0.05, Figure 2). The Bray–Curtis distances of relative abundances did not have significantly different dispersion across time, shape or polymer type (Betadisper ANOVA *p* for time = 0.99, shape = 0.71 and polymer type = 0.494). Bray–Curtis distances of relative abundance also had significantly different compositions across the environmental variables PAR, air temperature and dissolved oxygen (ADONIS *p* for all = 0.05). Sunken plastic did not show a significant difference between the bacterial community structure when compared to their still floating counterparts (ADONIS for time *p* = 0.69, location in tank = 0.235).

The prolific ‘early stage’ colonising bacterial taxa *Hyphomonas* is of particular interest due to its hydro‐carbon degrading capabilities (Denaro et al., 2020). That taxon remained the most dominant for the duration of the experiment with high relative abundance for the entire year, across plastic types and shapes (Figure 1), which we did not expect past the ‘early stage’ of colonisation. *Hyphomonas* is often highly enriched in hydrocarbon contaminated soils (Yakimov et al., 2005) and seas (Kappell et al., 2014) and is frequently associated with polycyclic aromatic hydrocarbons degradation (Wang et al., 2016). The second most dominant taxon in our experiment, *Qipengyuania*, was dominant towards the end of our experiment especially on PP (Figure 1). The known algicidal activity of this taxon may help explain the increase of this taxon over the experiment as the algal biofilm developed and strengthened (Ko et al., 2023). This taxon was also recently identified as an important taxon for the biosynthesis of intracellular microbial polyesters called polyhydroxyalkanoates (PHAs) from fish‐canning waste effluent (Correa‐Galeote et al., 2022). Further research is warranted to determine the potential for PHA production from plastic pollution in the marine environment even after the ‘early stage’ of bacterial colonisation on marine debris.

**FIGURE 3:**
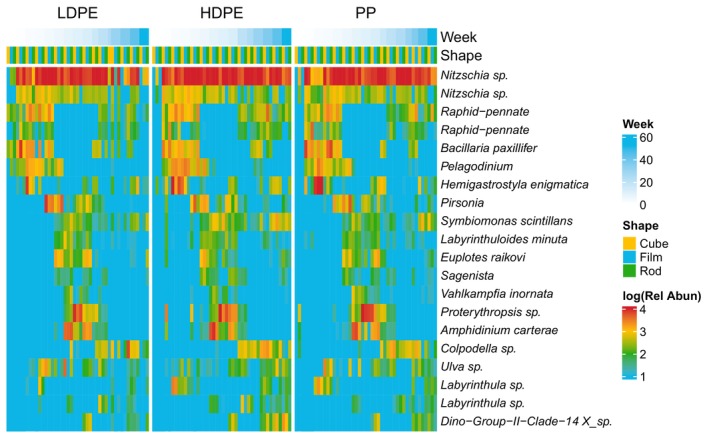
Heatmap of relative abundance (Rel Abun) of top 20 18S (eukaryotic), rRNA ASVs divided by polymer type (low‐density polyethylene (LDPE), high‐density polyethylene (HDPE) and polypropylene (PP)), shape (cube, film and rod) and ordered by week during the experiment from April 2021 to April 2022 in Hawai'i, USA.

The bacterial taxa *Rhodobacterales* and *Rosebacter* had high relative abundance at the same time as the ‘late‐stage’ diatom proliferation in the summer months (week 8–20, Figure 3) of the experiment. *Rhodobacterales* has been highlighted as a foundational member of the marine plastisphere, important for biofilm formation (Amaral‐Zettler et al., 2020; Zhai et al., 2023), and dependent on organic carbon substrates for growth (Koblizek, 2015). During diatom phytoplankton blooms in the open ocean, *Rhodobacterales* and *Roseobacter* are often the most dominant subgroups of bacteria present (Liu et al., 2013), demonstrating the complex interplay between eukaryotic and bacterial communities in both the ocean and on floating marine debris.

### Eukaryotic community structure

**FIGURE 4:**
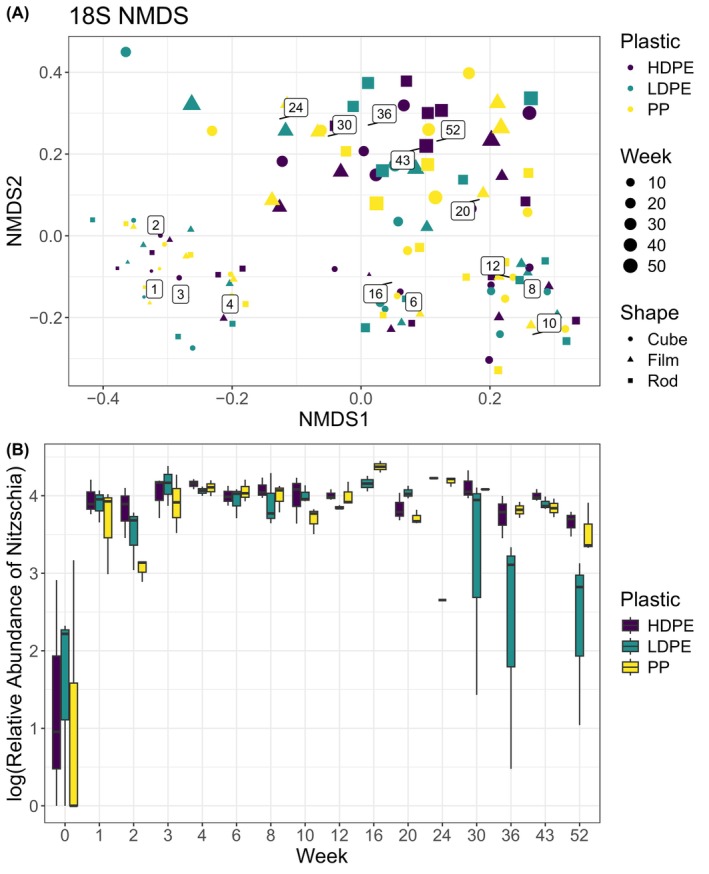
(A) Non‐metric multidimensional scaling (NMDS) of Bray–Curtis distance and (B) log(relative abundance) of the most dominant taxa *Nitzschia* over the year, where RA on low‐density polyethylene (LDPE) is significantly lower after week 24 (ANOVA *p* = 0.009). NMDS stress was 0.18, and labelled numbers are average values for week sampled. Community structure was not significantly different over time, shape or plastic type (ADONIS *p* for time = 0.94, shape = 0.95 and polymer type = 0.09).

Eukaryotic community structures (18S rRNA sequences) were dominated by the diatom *Nitzschia* across all plastic types and shapes for much of the year‐long experiment (Figure 3). Shannon diversity index was low for eukaryotic community structure and not significantly different across time or plastic shape (mean = 1.7, SD = 0.57, ANCOVA *p* shape = 0.06, type = 0.69), reflecting the dominance of *Nitzschia* on community structure. After week 30 however, *Nitzschia* was significantly lower on LDPE than other plastic types (ANOVA *p* = 0.009, Figure 4B).

In addition to *Nitzschia*, eukaryotic diversity was dominated by other diatoms for April to June 2021 (weeks 0–8), including two unclassified *Raphid‐pennate* diatom ASVs and *Bacillaria paxillifer*. Also, in those early weeks the dinoflagellate *Pelagodinum* and ciliate *Hemigastrostyla enigmatica* were present but less dominant across all plastic shapes and types. After June 2021 (week 8), other than the continued dominance of *Nitzchia*, the underlying eukaryotic community switched from primarily diatoms to other ciliates and heterotrophic dinoflagellates, including the especially dominant ciliate *Proterythropsis* and dinoflagellate *Amphidinium* (Figure 3). Despite this underlying change in community structure, eukaryotic microbial community structure was only slightly significantly different over time and not shape or polymer type in our NMDS analysis (ADONIS *p* for time = 0.05, shape = 0.09 and polymer type = 0.130, Figure 4A,B). Bray–Curtis distances of relative abundance had significantly different dispersion across time but not shape or polymer time (Betadisper ANOVA *p* for time = 1.1 × 10^−7^, shape = 0.87 and polymer type = 0.28). Bray–Curtis distances of relative abundance also had significantly different compositions across the environmental variables PAR, air temperature and dissolved oxygen (ADONIS *p* for all = 0.05). Finally, sunken plastic (1.02 m depth) did not have significantly different eukaryotic community structure when compared with their still floating counterparts (ADONIS *p* for time = 0.69, location = 0.235).

While Shannon eukaryotic diversity was not significantly different across time (ANOVA *p* = 0.8236), we saw a shift in the underlying eukaryotic taxa beginning in June 2021 (week 8), when temperatures began to rise. The anomalously high oxygen leading to early November (week 30) occurred at the same time as a change in the most dominant eukaryotic taxa, with *Nitzschia* decreasing in relative abundance on all plastics, and decreasing significantly on LDPE. A decrease in the dominant eukaryotic taxa of a mature plastic biofilm is less commonly reported in other studies, as most plastic incubation studies are <1 month and most do not even include eukaryotic diversity (Wright et al., 2020). Longer‐term incubations (years) are necessary to elucidate differences in eukaryotic species and their bacterial associates on mature plastisphere biofilms.

### Differential abundance across PP and PE

**FIGURE 5:**
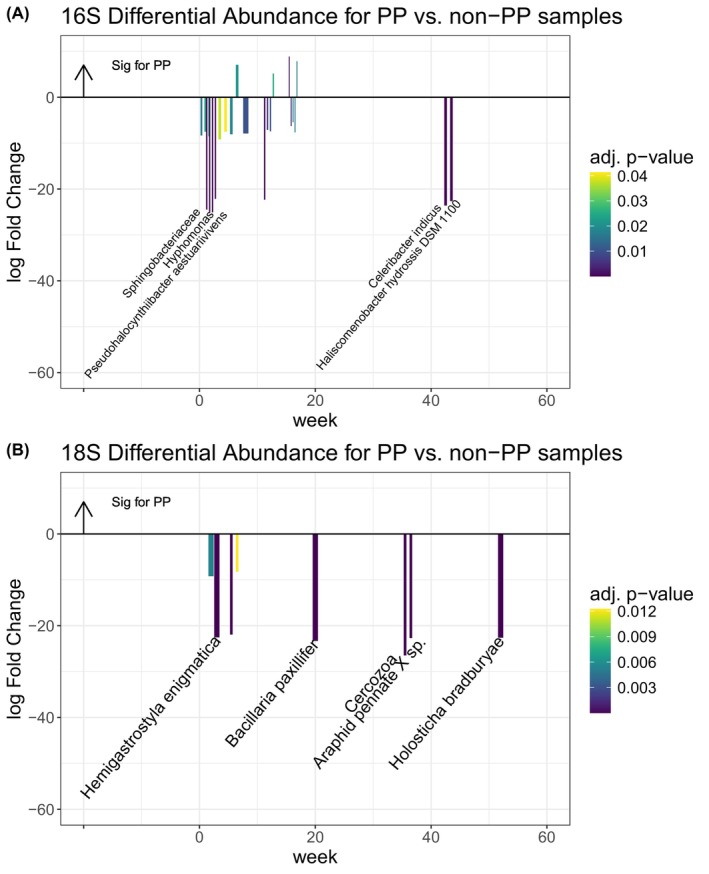
Deseq2 results for significantly differentially abundant genera across time, where the five most differential genera are labelled. Positive log change are genera that are more highly abundant in PP samples (indicated by arrow), and Negative log change are genera that are more highly abundant in PE samples for (A) 16S and (B) 18S during the experiment from April 2021 to April 2022 in Hawai'i, USA.

Our differential abundance analysis, which demonstrates which microbes are more highly abundant on different plastics, demonstrated that plastic degraders may colonise PE faster than PP during ‘early stage’ colonisation. A total of 24 bacterial taxa and eight eukaryotic taxa were significantly differentially abundant (*p* < 0.05, with exact values in Figure S2) for the polymers over the 15 timepoints (deseq2 adj *p* < 0.05 for PP vs. PE samples tested at each timepoint independently). Most (87%) of these bacterial taxa and all (100%) of these eukaryotic taxa were more abundant in PE samples when compared with PP samples (demonstrating a negative log Fold change in Figure 5). Most of the bacteria taxa that were significantly differentially abundant were discovered at timepoints before week 20 (Figure 5A), while significantly differentially abundant eukaryotic taxa were more evenly spread throughout timepoints over the year (Figure 5B). The chemical differences across PE and PP are demonstrated in the companion paper Brignac et al., in prep., which includes a full chemical analysis of this experiment.

The three most differentially abundant taxa on PE during the experiment all occurred in week two and were the hydro‐carbon degraders *Hyphomonas*, *Sphingobacteriaceae* and *Pseudohalocynthiibacter aestuariivivens*. In a recent study, a *Pseudohalocynthiibacter* from a highly polluted coastal environment demonstrated enhanced biosynthesis of PHAs from the breakdown of glucose (Esposito et al., 2023). The highly significant differential abundance of these taxa demonstrates that plastic degraders may colonise PE more robustly than PP during ‘early stage’ colonisation. For the eukaryotes, the presence of the most significantly differentially abundant taxa on PE—the marine ciliate *Holosticha bradburyae* at week 52—aligns with our findings of decreased diatom presence on LDPE after week 40. Overall, our findings indicate longer incubation times (>1 month) are necessary to demonstrate changes in eukaryotic diversity on marine plastic debris.

## AUTHOR CONTRIBUTIONS

**Elizabeth Connors:** Conceptualization (equal); data curation (equal); formal analysis (equal); methodology (equal); writing – original draft (equal); writing – review and editing (equal). **Laurent Lebreton:** Conceptualization (equal); funding acquisition (equal); project administration (equal); writing – review and editing (equal). **Jeff S. Bowman:** Conceptualization (equal); investigation (equal); project administration (equal); supervision (equal); writing – review and editing (equal). **Sarah‐Jeanne Royer:** Conceptualization (equal); data curation (equal); funding acquisition (equal); investigation (equal); methodology (equal); project administration (equal); resources (equal); supervision (equal); writing – review and editing (equal).

## CONFLICT OF INTEREST STATEMENT

The authors declare no conflicts of interest.

## Supporting information


**Figure S1.** Daytime averages of environmental data of PAR (photosynthetically active radiation, μmol·m^−2^·s^−1^, A), dissolved oxygen (mg L^−1^, B), water temperature (°C, C) and air temperature (°C, D) in the experiment from April 2021 to April 2022 in Hawai'i, USA. Points are average data leading up to sampling events, with dashed lines connecting.

**Figure S2.** Deseq2 results for significantly different abundance across time. Includes week tested, kingdom and genera, adjusted p‐value and log change in differential abundance. Positive log change are taxa that are more highly abundant in PP samples, and Negative log change are taxa that are more highly abundant in PE samples.

## Data Availability

All sequences were submitted to NCBI BioProject PRJNA 1020163. Code and data repository for this manuscript is located on the first author's GitHub, at https://github.com/beth-connors/OceanCleanup.
