# Supplementary figures and images for: Changes in microbial community structure of bio‐fouled polyolefins over a year‐long seawater incubation in Hawai'i
Source: Environ Microbiol Rep. 2024 Jul 29;16(4):e13283. doi: 10.1111/1758-2229.13283 (PMC11286543; doi:10.1111/1758-2229.13283)

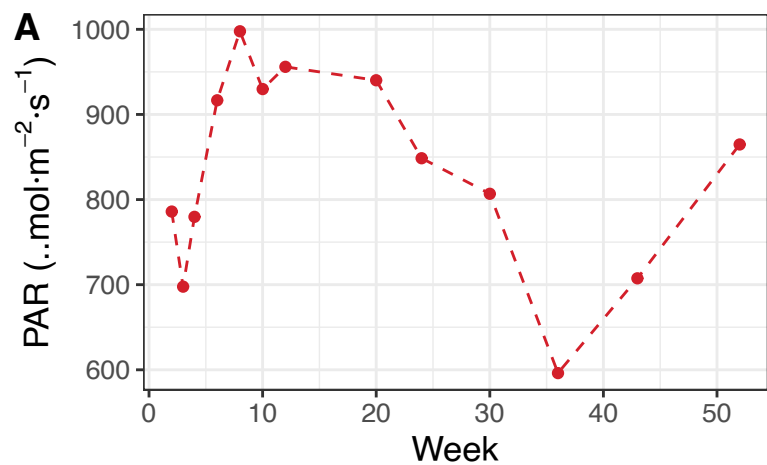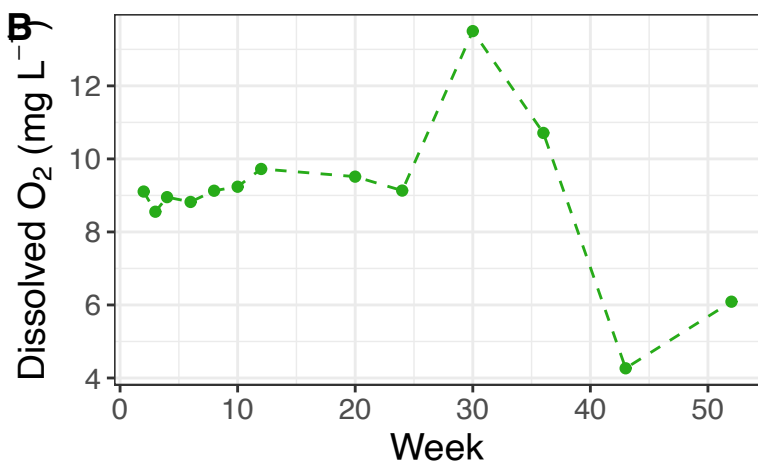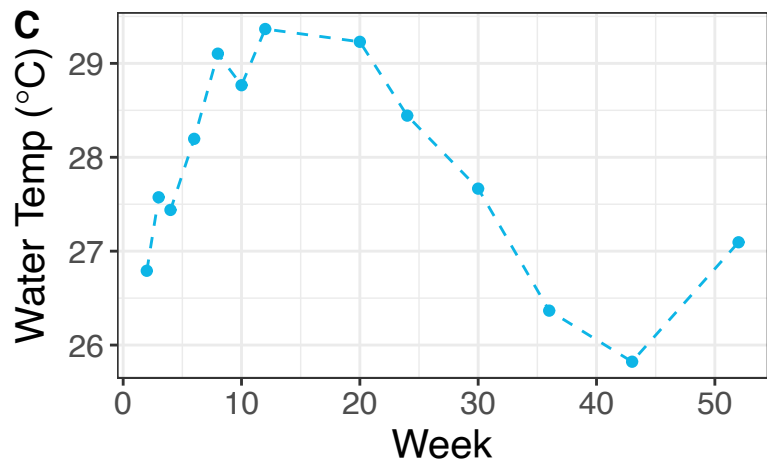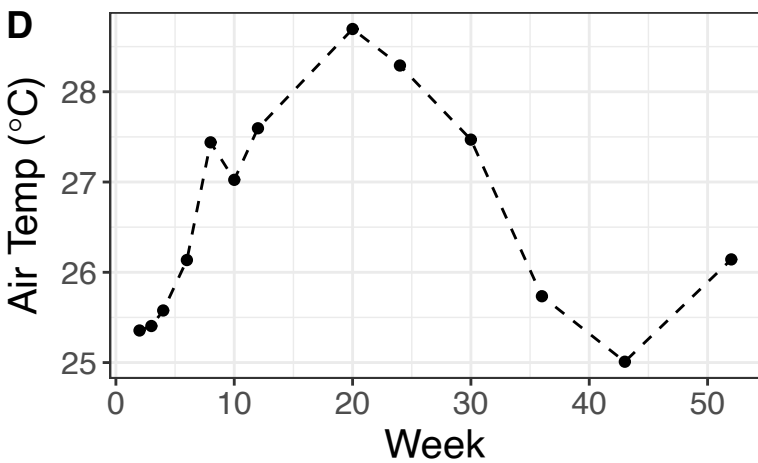

Supplement: Supplementary file 1 — Figure S1. Daytime averages of environmental data of PAR (photosynthetically active radiation, μmol·m−2·s−1, A), dissolved oxygen (mg L−1, B), water temperature (°C, C) and air temperature (°C, D) in the experiment from April 2021 to April 2022 in Hawai'i, USA. Points are average data leading up to sampling events, with dashed lines connecting. [file EMI4-16-e13283-s001.pdf]
